# Neuroprotective effect of 5-aminolevulinic acid against low inorganic phosphate in neuroblastoma SH-SY5Y cells

**DOI:** 10.1038/s41598-017-06406-6

**Published:** 2017-07-18

**Authors:** Naoko Takase, Masatoshi Inden, Shin-ichiro Sekine, Yumi Ishii, Hiroko Yonemitsu, Wakana Iwashita, Hisaka Kurita, Yutaka Taketani, Isao Hozumi

**Affiliations:** 1 Laboratory of Medical Therapeutics and Molecular Therapeutics, Gifu Pharmaceutical University, Gifu, Japan; 2 Department of Clinical Nutrition and Food Management, Institute of Biomedical Sciences, Tokushima University Graduate School, Tokushima, Japan

## Abstract

PiT-1 (encoded by *SLC20A1*) and PiT-2 (encoded by *SLC20A2*) are type-III sodium-dependent phosphate cotransporters (NaPiTs). Recently, *SLC20A2* mutations have been found in patients with idiopathic basal ganglia calcification (IBGC), and were predicted to bring about an inability to transport Pi from the extracellular environment. Here we investigated the effect of low Pi loading on the human neuroblastoma SH-SY5Y and the human glioblastoma A172 cell lines. The results show a different sensitivity to low Pi loading and differential regulation of type-III NaPiTs in these cells. We also examined whether 5-aminolevulinic acid (5-ALA) inhibited low Pi loading-induced neurotoxicity in SH-SY5Y cells. Concomitant application of 5-ALA with low Pi loading markedly attenuated low Pi-induced cell death and mitochondrial dysfunction *via* the induction of HO-1 by p38 MAPK. The findings provide us with novel viewpoints to understand the pathophysiology of IBGC, and give a new insight into the clinical prevention and treatment of IBGC.

## Introduction

Inorganic phosphate (Pi) is an essential nutrient for a variety of biological functions, such as cell signaling, nucleic acid synthesis, energy metabolism, membrane function, as well as bone mineralization. Pi is required for optimal cellular growth, and homeostatic mechanisms exist to maintain extra- and intracellular Pi content within the physiologic range through the regulatory actions of sodium-dependent phosphate cotransporters (NaPiTs). There are three families of NaPiTs: type-I (the SLC17 family), type-II (the SLC34 family), and type-III (the SLC20 family)^1^. Among them, PiT-1 (encoded by *SLC20A1*) and PiT-2 (encoded by *SLC20A2*) are type-III NaPiTs. Type-III NaPiTs have a broad tissue distribution, suggesting they play a housekeeping role for Pi homeostasis in mammalian cells^2–4^. The contribution of Pi to the regulation of mitochondrial respiratory control and mitochondrial substrate oxidation has also been reported in previous studies^5, 6^. Pesta *et al*. suggested that Pi is an important determinant of mitochondrial adenosine 5′-triphosphate (ATP) synthesis *via* mitochondrial phosphorylation activity^7^. The enzyme responsible for ATP synthesis is associated with the Pi carrier and ADP carrier in a large protein complex called ATP synthasome. ATP synthase then combines Pi and ADP to form ATP^8^. Therefore, an increase in intracellular Pi content is likely to activate ATP synthesis, and conversely, a decrease in intracellular Pi content may lower ATP synthesis and subsequently lead to mitochondrial dysfunction and cell damage.

Familial idiopathic basal ganglia calcification (IBGC) is a rare genetic condition characterized by symmetric calcification in the basal ganglia and other brain regions, and a wide spectrum of neuropsychiatric symptoms^9^. In 2012, the first causative gene was determined to be *SLC20A2*, which was linked to IBGC by the identification of seven families with IBGC that had mutations in this gene^10^. In addition, several groups, including our own research team, have reported novel mutations in *SLC20A2* ^11–15^. Mutations of *SLC20A2* associated with IBGC were shown or were predicted to result in an inability to transport Pi from the extracellular environment^10–15^. For instance, Wang *et al*. showed that the six mutations of *SLC20A2* in IBGC patients (encoding S601W, S601L, T595M, E575M, G498R, and V42del) brought about impaired Pi uptake using ^32^Pi transport assays in *Xenopus oocytes* ^10^. Yamada *et al*. showed that *in silico* analysis using PolyPhen-2 of the missense mutations of *SLC20A2* (encoding T115M and S637R) predicted that all were likely to be damaging to the function of *SLC20A2* ^12^. Therefore, at least, mutations in *SLC20A2* lead to insufficient Pi uptake capability. These results suggest that the mutations in *SLC20A2* may induce depletion of intracellular Pi.

5-Aminolevulinic acid (5-ALA), a natural amino acid, is synthesized through the condensation of glycine and succinyl-CoA by the catalytic effect of 5-ALA synthase. In the cytosol, 5-ALA is converted into coproporphyrinogen III by sequential biosynthetic pathways *via* several intermediates (beginning with 5-ALA, and through porphobilinogen, hydroxymethylbilane, and uroporphyrinogen III, before being finally converted to coproporphyrinogen III). Coproporphyrinogen III is transported to the mitochondria and it undergoes further biosynthesis, being converted to protoporphyrin IX *via* the intermediate, protoporphyrinogen IX. Iron is inserted into protoporphyrin IX *via* a ferrochelatase-catalyzed reaction to eventually generate heme^16–18^. Heme oxygenase-1 (HO-1) is a key enzyme for heme metabolism. HO-1 metabolizes excess heme, which can cause cell toxicity, and protects cells from oxidative stress^19^. Recent studies have reported that 5-ALA induces the upregulation of HO-1^16–18^. In addition, 5-ALA has been implicated in the treatment of inflammatory disease, autoimmune disease and transplantation due to the anti-inflammatory and immunoregulatory actions that are associated with the expression of HO-1 *via* mitogen-activated protein kinase (MAPK) activation^16–18^.

Recently, we have detected type-III NaPiTs immunopositivity in neurons, astrocytes, and vascular endothelial cells^20, 21^. The results suggest that Pi homeostasis may be controlled by different mechanisms underlying activation of PiT-1 and PiT-2 in each cell type, and also, that the degree of cellular dysfunction and/or cytotoxicity is different even in the CNS cells in which both PiT-1 and PiT-2 are expressed^20, 21^. In addition, mutations of *SLC20A2* associated with IBGC were predicted to induce depletion of intracellular Pi^10–15^. However, the role of type-III NaPiTs in CNS cells is still unclear. Here, we investigated the effect of low Pi loading on the human neuroblastoma cell line SH-SY5Y and the human glioblastoma cell line A172. In addition, we examined whether 5-ALA inhibited low Pi loading-induced neurotoxicity *via* the induction of HO-1 by MAPK activation in SH-SY5Y cells.

## Results

### Effects of low Pi loading on cytotoxicity

**Figure 1:**
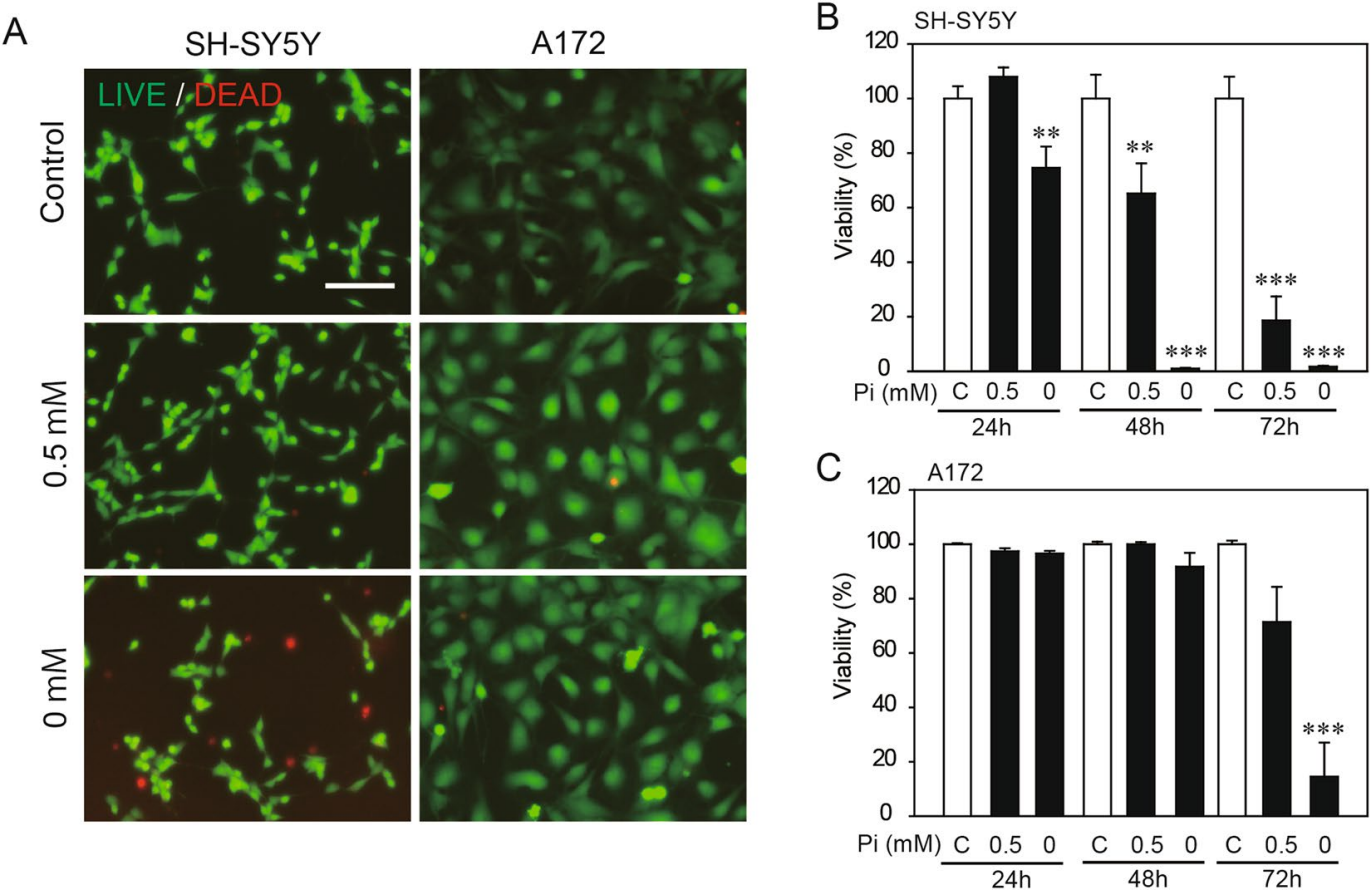
Effects of low Pi loading on cytotoxicity in SH-SY5Y or A172 cells. SH-SY5Y or A172 cells were cultured overnight in FBS-free/Pi-free DMEM. We then added appropriate amounts of sodium phosphate buffer to produce final Pi concentrations of 0, 0.5, and 1 mM (control concentration). For the cytotoxicity assays, the LIVE/DEAD Cell Imaging Kit (**A**) and Cell Count Reagent SF (**B**,**C**) were used in accordance with the manufacturer’s instructions. Data are represented as the mean ± SEM of three determinations, based on treatment with 1 mM Pi giving a viability of 100%. The significance of any difference was determined using ANOVA followed by the *post-hoc* Bonferroni/Dunn test (**p < 0.01, ***p < 0.001). Scale bar, 75 µm in (**A**).

At 24 h after low Pi loading, SH-SY5Y cells treated with 0 mM Pi exhibited cell death, but those treated with 0.5 and 1 mM Pi did not (Fig. 1A left lane). On the other hand, A172 cells treated with 0, 0.5 and 1 mM Pi did not exhibit cell death at 24 h after low Pi loading (Fig. 1A right lane). Treatment with 0 mM Pi for 48 h completely caused cell death in SH-SY5Y cells (Fig. 1B). Long-term treatment with 0.5 mM Pi (for 24, 48, and 72 h) increased death of SH-SY5Y cells in a time-dependent manner (Fig. 1B). However, in A172 cells, significant cell death was observed only at 72 h after 0 mM Pi loading, and no other low Pi loading treatment-induced cell death during our experimental period (Fig. 1C).

### Effects of low Pi loading on mRNA expression of type-III NaPiTs

**Figure 2:**
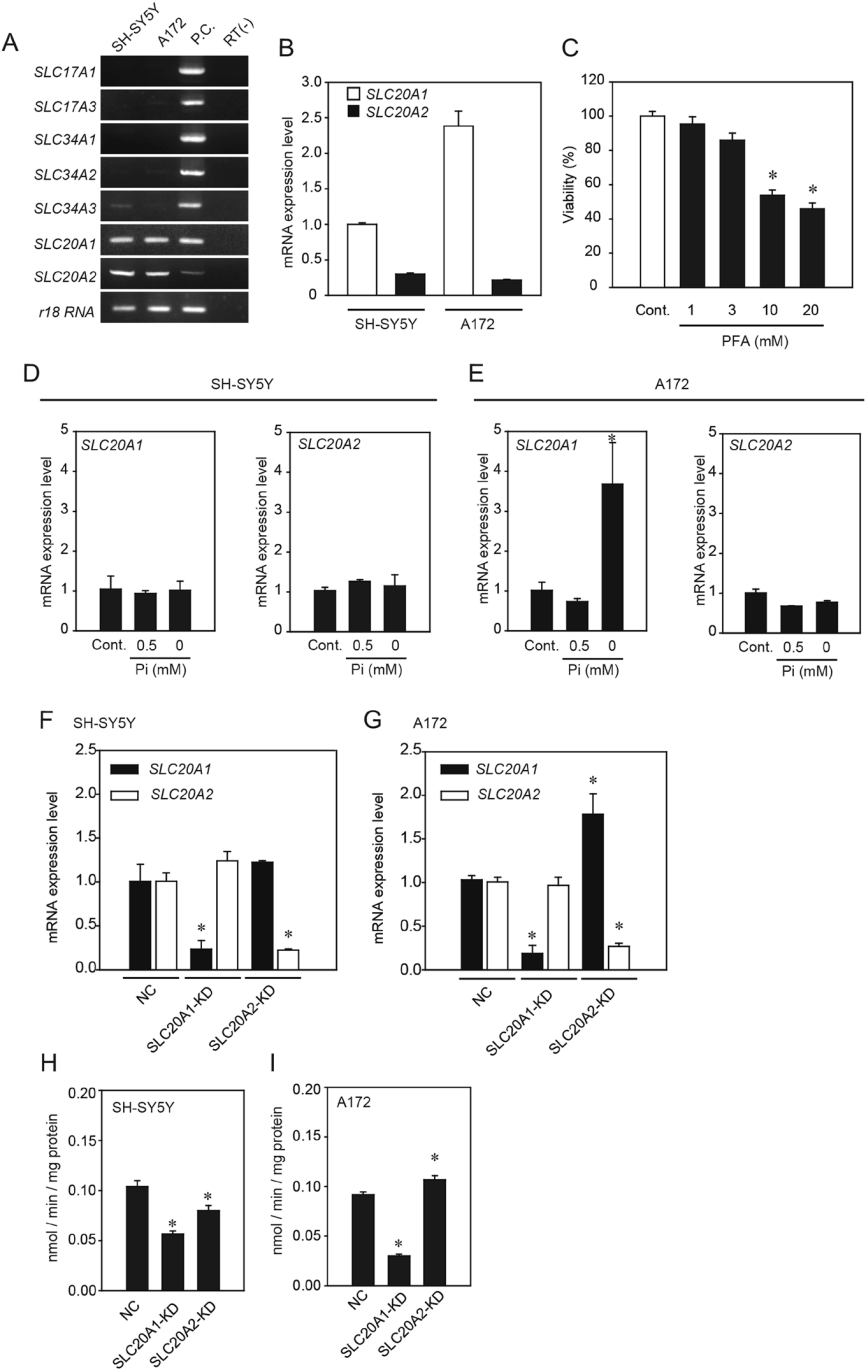
Effects of low Pi loading on type-III NaPiT mRNA expression. cDNA reverse transcribed from the mRNA of SH-SY5Y or A172 cells was used as a template for the RT-PCR and qRT-PCR assays. (**A**) Expression of NaPiT mRNA in SH-SY5Y or A172 cells. For each amplification reaction, positive control reactions (PC) were performed using cDNA templates from the kidney (except for *SLC34A2*) or lung (for *SLC34A2*). Negative control reactions lacking RT reactions (RT-) were also performed to exclude the possibility of genomic DNA contamination. The sequences of primer sets are shown in Table 1. (**B**) The TaqMan-based qPCR assay using *SLC20A1* and *SLC20A2* probes. Data were normalized to the amount of *18s ribosomal RNA* (*18s rRNA*), and results are expressed as the fold increase compared with that in the SH-SY5Y cells (mean ± SEM; *n* = 3). (**C**) Effect of PFA on cytotoxicity. (**D**,**E**) At 24 h after low Pi loading treatment, mRNA expressions of *SLC20A1* and *SLC20A2* was analyzed using the TaqMan-based qPCR assay. (**F**,**G**) SH-SY5Y and A172 cells were transiently transfected with *SLC20A1* or *SLC20A2* siRNA. At 24 h after transfection, mRNA expressions of *SLC20A1* and *SLC20A2* were analyzed using the TaqMan-based qPCR assay. (**H**,**I**) The Pi uptake assay was carried out in *SLC20A1*- and *SLC20A2*-suppressed cells. Data were normalized to the amount of *18s rRNA*, and results are expressed as the fold increase compared with that at 1 mM Pi (**D**,**E**) or in NC (**F**–**I**) (mean ± SEM; *n* = 3). The significance of any difference was determined using ANOVA followed by the Bonferroni/Dunn *post-hoc* test (*p < 0.05).

Previous studies have shown that changes in extracellular Pi content modified the expression of NaPiTs mRNA including that of *SLC20A1* and *SLC20A2* ^2, 22, 23^. Therefore, we postulated that low Pi loading could change mRNA expressions of *SLC20A1* and *SLC20A2* in neurons and glial cells, and we determined which NaPiTs are expressed in SH-SY5Y and A172 cells (Fig. 2A). Reverse transcription polymerase chain reaction (RT-PCR) analysis showed that *SLC20A1* and *SLC20A2* mRNAs were clearly expressed in both SH-SY5Y and A172 cells. The mRNA of other NaPiTs including *SLC17A1*, *SLC17A3*, *SLC34A1*, *SLC34A2*, and *SLC34A3* was barely detected in these cells in our experimental conditions (Fig. 2A). Quantitative RT-PCR (qRT-PCR) analysis showed that the expression level of *SLC20A1* mRNA was higher than the expression level of *SLC20A2* mRNA in both SH-SY5Y and A172 cells (Fig. 2B). In addition, phosphonoformic acid (PFA), an inhibitor of PiT-1 and PiT-2, induced cell death in a concentration-dependent manner, suggesting that PiT-1 and PiT-2 play a functional role in cell viability in SH-SY5Y and A172 cells (Fig. 2C).

In SH-SY5Y cells, mRNA expressions of *SLC20A1* and *SLC20A2* were not affected by low Pi loading for 24 h (Fig. 2D). On the other hand, *SLC20A1* mRNA expression in A172 cells was significantly increased by treatment with 0 mM Pi for 24 h, although *SLC20A2* mRNA expression did not change (Fig. 2E). To confirm the compensatory role of PiT-1 and PiT-2, we also performed a knockdown experiment using siRNA against *SLC20A1* and *SLC20A2* in SH-SY5Y cells and A172 cells. We found that in SH-SY5Y cells, *SLC20A1* mRNA expression was not changed by the suppression of *SLC20A2* expression, compared with non-targeted siRNA controls (NC) (Fig. 2F). In A172 cells, *SLC20A1* mRNA expression was significantly elevated in *SLC20A2-*suppressed cells (Fig. 2G). On the other hand, *SLC20A2* mRNA expression was not changed by the suppression of *SLC20A1* mRNA expression in either SH-SY5Y cells or A172 cells (Fig. 2F and G).

In SH-SY5Y cells, Pi uptake was significantly decreased by the suppression of either *SLC20A1* or *SLC20A2* expression, compared with NC (Fig. 2H). However, although Pi uptake was significantly decreased by the suppression of *SLC20A1* expression in A172 cells, Pi uptake was significantly increased by the suppression of *SLC20A2* expression (Fig. 2I).

### Effects of 5-ALA on cytotoxicity induced by low Pi loading

**Figure 3:**
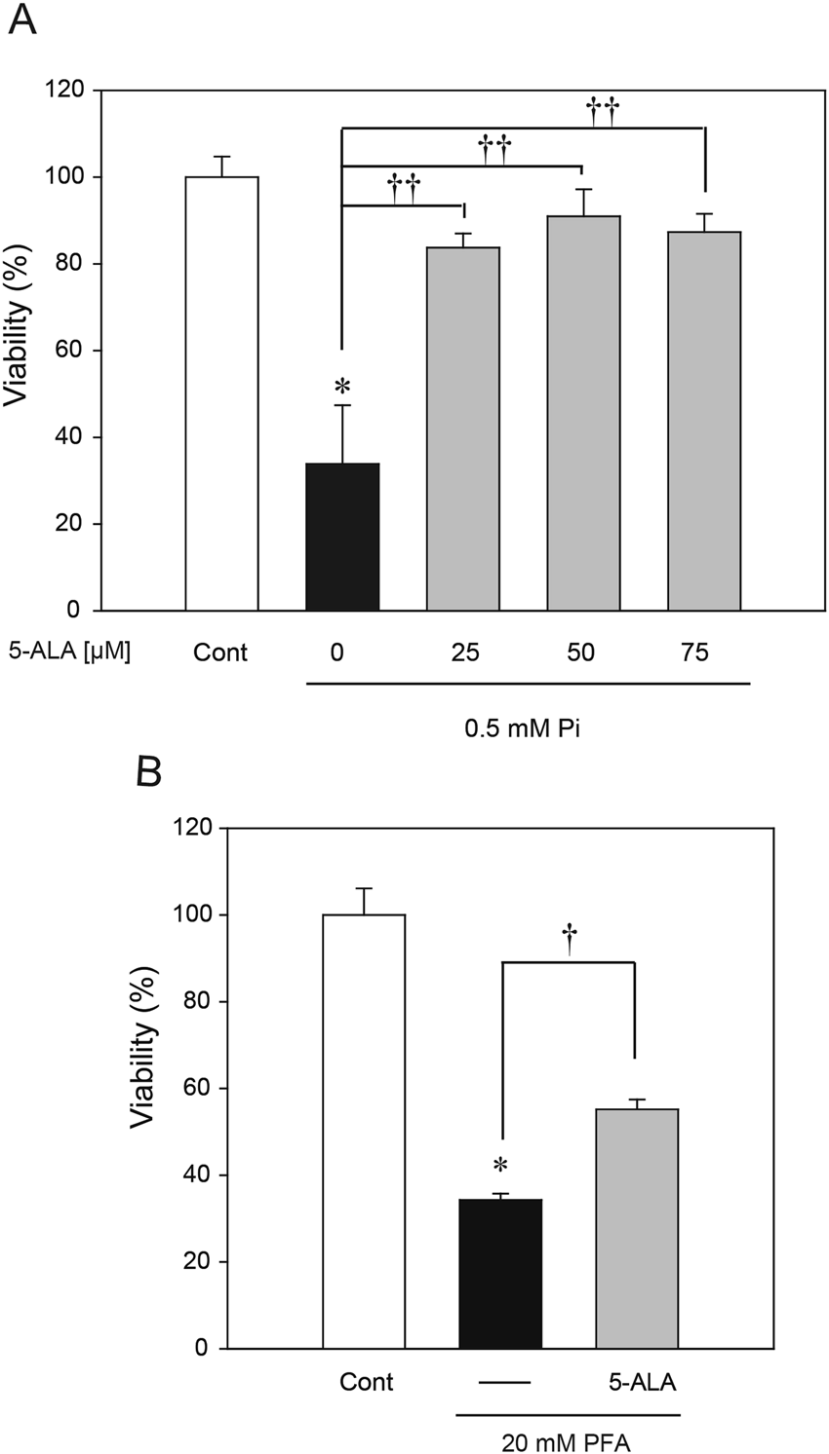
Effects of 5-ALA on low Pi-induced cell death. (**A**) MTT assay for cell survival at 48 h after simultaneous treatment with 0.5 mM Pi in the absence or presence of 5-ALA (at 25, 50, 75 µM). (**B**) MTT assay for cell survival at 24 h after simultaneous treatment with 20 mM PFA in the absence or presence of 50 µM 5-ALA. Each value represents the mean ± SEM of three determinations, based on the viability of the untreated culture being 100% (control). The significance of any difference was determined using ANOVA, followed by the Bonferroni/Dunn *post-hoc* test (*p < 0.05 versus control; ^†^ *p* < 0.05, ^††^ *p* < 0.01 versus treatment with 0.5 mM Pi or 20 mM PFA alone).

In order to examine whether 5-ALA could inhibit low Pi-induced cell death in SH-SY5Y cells, we performed a 3-(4,5-dimethyl-2-thiazolyl)-2,5-diphenyltetrazolium bromide (MTT) assay. Concomitant application of 5-ALA (25–75 µM) with low Pi loading markedly attenuated low Pi-induced cell death (Fig. 3A). Similarly, treatment with 50 µM 5-ALA significantly attenuated PFA-induced cell death (Fig. 3B).

We also examined the involvement of mRNA expressions of *SLC20A1* and *SLC20A2* in 5-ALA-induced neuronal protection. 5-ALA had no effect on mRNA expressions of *SLC20A1* and *SLC20A2* (Supplementary Fig. S1).

### Effects of 5-ALA on mitochondrial dysfunction and oxidative stress induced by low Pi loading

**Figure 4:**
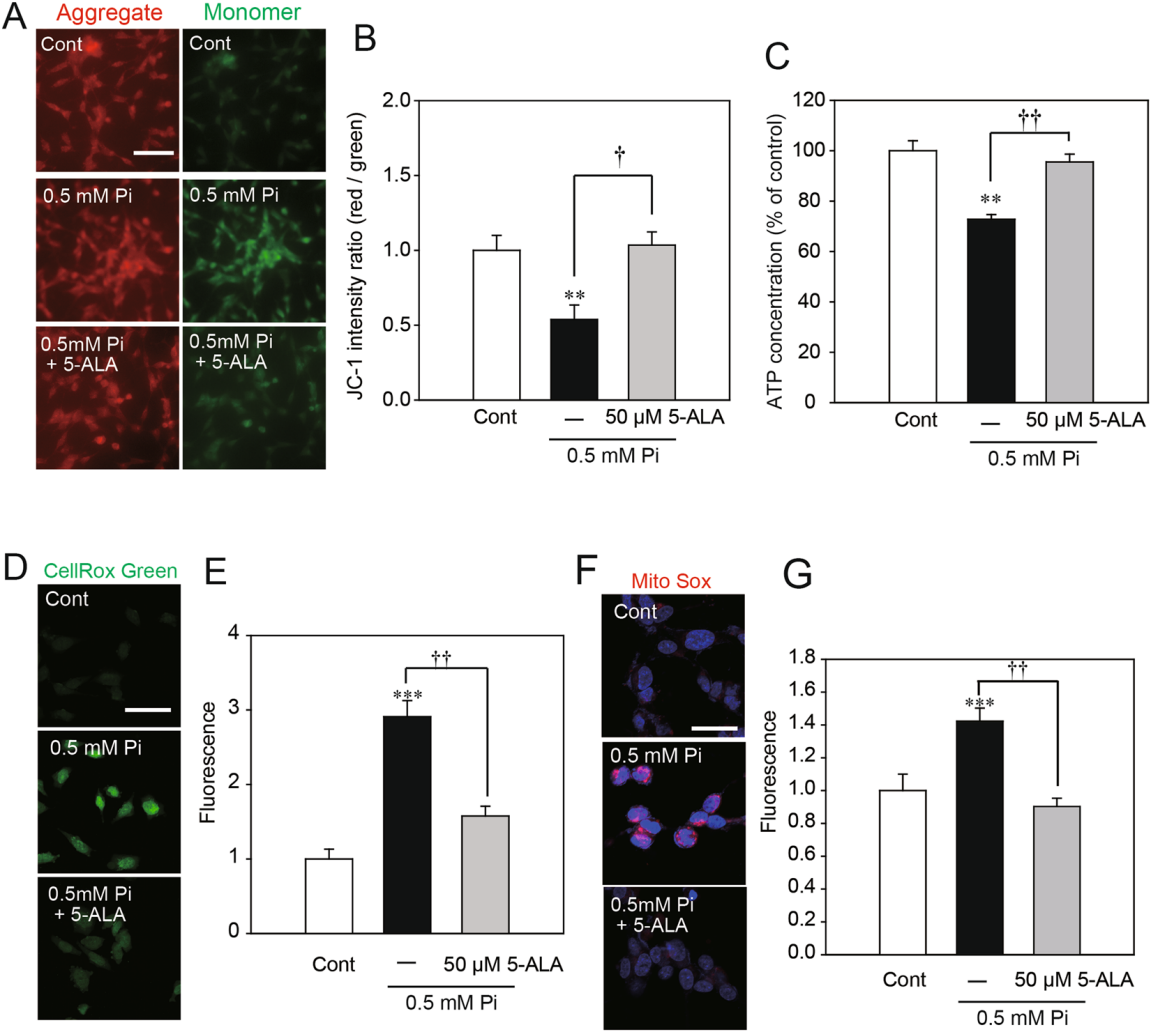
Effects of 5-ALA on mitochondrial membrane potential loss and oxidative stress induced by low Pi. (**A**,**B**) The density of JC-1 staining was assessed by a fluorescence microscopy system. (**C**) Measurement of cellular ATP content. The densities of the CellROX dye (**D**,**E**) and the MitoSOX dye (**F**,**G**) were assessed by a fluorescence microscopy system. Each value represents the mean ± SEM of three determinations, based on the untreated culture (control). The significance of any difference was determined using ANOVA, followed by the Bonferroni/Dunn *post-hoc* test (*p < 0.05 versus control; ^†^ *p* < 0.05, ^††^ *p* < 0.01 versus treatment with 0.5 mM Pi alone). Scale bar, 20 µm in (**A**,**D**,**F**).

The contribution of Pi to the regulation of mitochondrial respiratory control and mitochondrial substrate oxidation has been reported previously^5, 6^. An earlier study showed that mitochondrial dysfunction caused by high Pi levels induced the production of reactive oxygen species (ROS) in bovine aortic smooth muscle cells^24^. Thus, we examined whether low Pi levels could affect the regulation of the mitochondrial membrane potential (MP) and the production of ROS. Representative images show that the ratio of mitochondrial JC-1 aggregates (red) to cytosolic JC-1 monomers (green) was reduced 24 h after low Pi loading, compared with the control condition (Fig. 4A and B). In addition, low Pi loading reduced ATP synthesis (Fig. 4C). To investigate whether low Pi loading could increase ROS production in SH-SY5Y cells, all control and treatment groups were examined using the CellROX dye, where changes in fluorescence intensity act as an indicator of ROS production. As shown in Fig. 4D and E, low Pi loading significantly enhanced ROS production. As the main source of cellular ROS production is mitochondrial dysfunction, we further measured mitochondrial O_2_^−^ using the MitoSOX dye. Low Pi loading significantly increased MitoSOX fluorescence intensity (Fig. 4F and G).

We also investigated whether 5-ALA could prevent mitochondrial dysfunction and ROS production caused by low Pi loading. 5-ALA improved the reduction in the red/green fluorescence intensity ratio of JC-1 after low Pi loading (Fig. 4A and B) and recovered ATP synthesis (Fig. 4C). Moreover, 5-ALA significantly decreased the low Pi loading-induced enhancement of mitochondrial O_2_^−^ levels, and the subsequent heightened oxidative stress was significantly alleviated (Fig. 4D–G).

### 5-ALA increases HO-1 up-regulation *via* p38 MAPK

**Figure 5:**
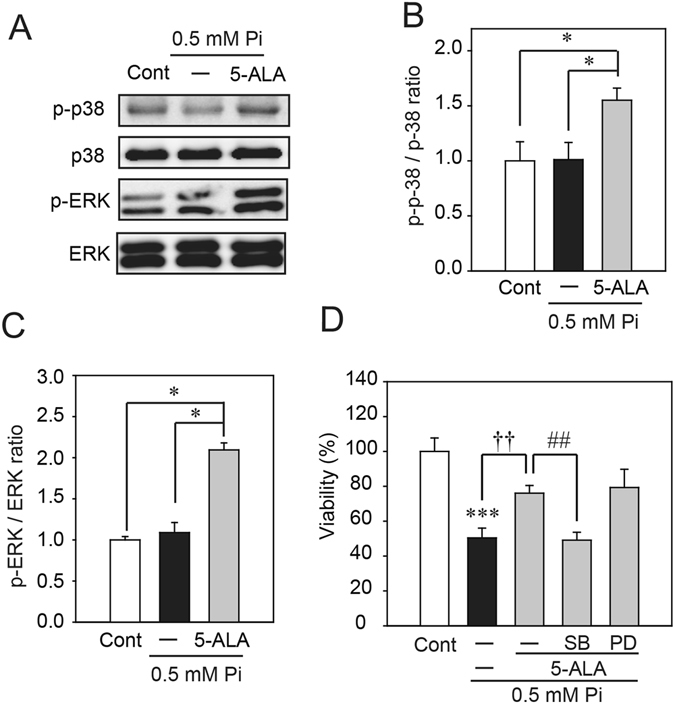
5-ALA activation of the MAPK signaling pathway. (**A**–**C**) Western blot analysis of the effects of 5-ALA on the phosphorylation levels of p38 ERK 1/2 MAPK. (**D**) Effect of MAPK inhibitors on the protective effect of 5-ALA on low Pi-induced cell death. SB203580 (SB), PD98059 (PD), and 5-ALA were applied with 0.5 mM Pi for 1 h (**A**–**C**) and 48 h (**D**). Each value represents the mean ± SEM of three determinations, based on the untreated culture (control). The significance of any difference was determined using ANOVA, followed by the Bonferroni/Dunn *post-hoc* test (*p < 0.05 versus control; ^†^ *p* < 0.05, ^††^ *p* < 0.01 versus treatment with 0.5 mM Pi alone).

We examined the involvement of MAPK cascades leading to the activation of ERK and p38, as 5-ALA has been shown to activate ERK and p38^16^. As shown in Fig. 5A–C, treatment with 5-ALA for 1 h activated ERK and p38. Concomitant application of SB203580 (SB), a p38 inhibitor, with low Pi loading for 48 h attenuated the protective effect of 5-ALA on low Pi loading-induced cell death in SH-SY5Y cells (Fig. 5D). On the other hand, concomitant application of PD98059 (PD), an ERK inhibitor, did not have an effect (Fig. 5D).

**Figure 6:**
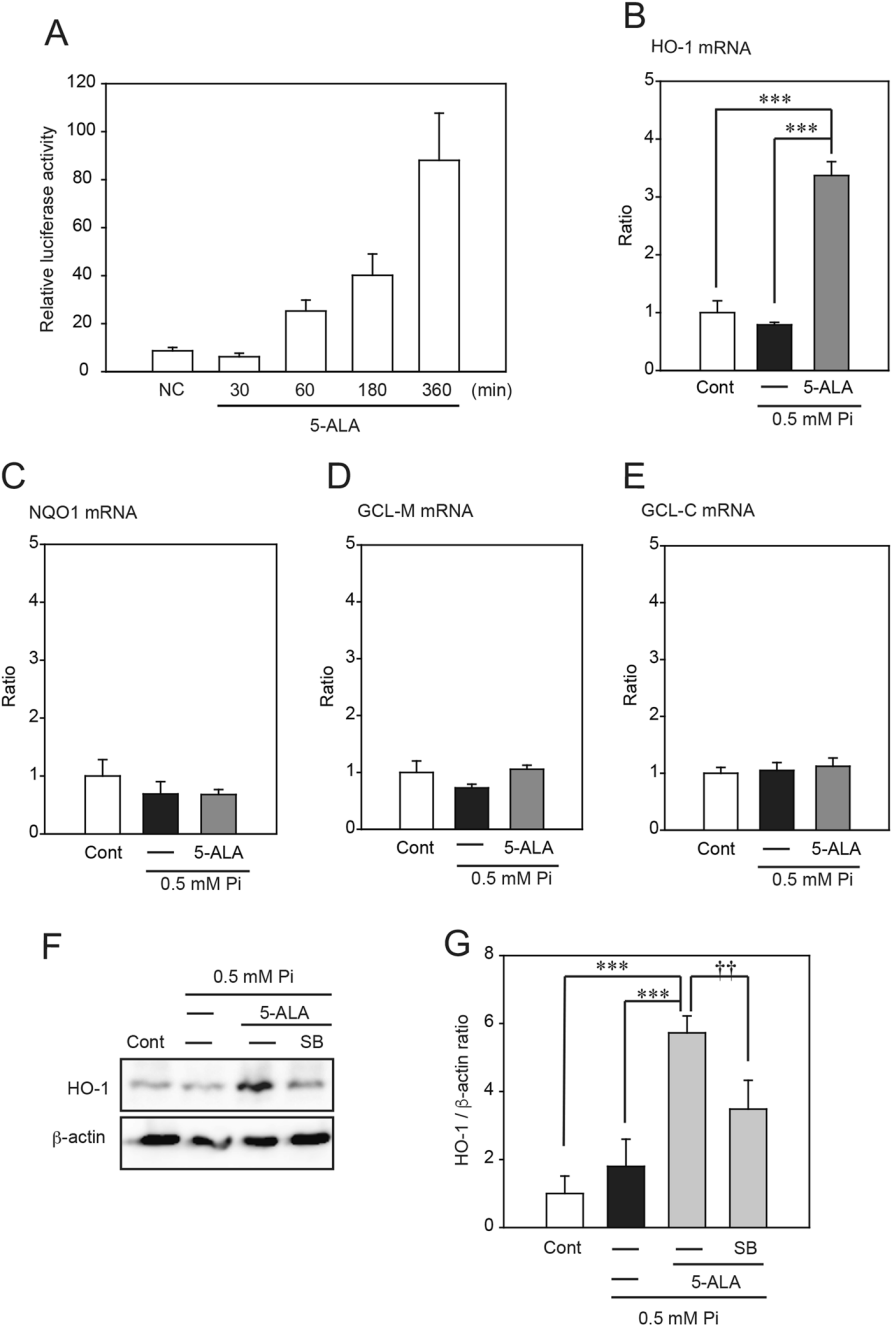
The p38 MAPK signaling pathway is involved in the increased expression of HO-1 induced by 5-ALA. (**A**) The ARE reporter assay. (**B**–**E**) qRT-PCR analysis of the effects of 5-ALA on the mRNA expression level of HO-1 (**B**), NQO1 (**C**), GCLM (**D**), and GCLC (**E**). (**F**,**G**) Western blot analysis showing the effect of the p38 MAPK inhibitor SB203580 (SB) on 5-ALA-induced expression of HO-1. Each value represents the mean ± SEM of three determinations, based on the untreated culture (control). The significance of any difference was determined using ANOVA, followed by the Bonferroni/Dunn *post-hoc* test (*p < 0.05 versus control; ^†^ *p* < 0.05, ^††^ *p* < 0.01 versus treatment with 0.5 mM Pi).

In a previous study 5-ALA was shown to induce activation of nuclear factor erythroid 2-related factor 2 (Nrf2) *via* MAPK^16^. Nrf2 induces the expression of antioxidant and phase II enzymes by binding to the antioxidant response element (ARE) region of the gene promoter. To examine whether the actions of 5-ALA contribute to the Nrf2 antioxidant pathway, we carried out an ARE reporter assay (Fig. 6A). 5-ALA induced luciferase activity, suggesting that it contributes to the Nrf2 antioxidant response pathway. Nrf2 regulates the expression of antioxidative and phase II detoxifying enzymes including HO-1, NAD(P)H:quinone oxidoreductase 1 (NQO1), γ-glutamyl cysteine synthetase modifier subunit (GCLM), and γ-glutamyl cysteine synthetase catalytic subunit (GCLC). qRT-PCR analysis showed that *HO-1* mRNA was increased at 12 h by 5-ALA (Fig. 6B). However, other enzymes were not affected (Fig. 6C–E). Western blot analysis also demonstrated that levels of the HO-1 protein were increased at 24 h after 5-ALA treatment (Fig. 6F). Moreover, concomitant application of SB with low Pi loading attenuated 5-ALA-induced HO-1 expression (Fig. 6G).

## Discussion

We have found that SH-SY5Y cells are more vulnerable upon exposure to low Pi levels compared with A172 cells. In SH-SY5Y cells, mRNA expressions of *SLC20A1* and *SLC20A2* were not affected by low Pi loading. Similarly, expressions of these mRNA were not affected by siRNA transfection of *SLC20A2* or *SLC20A1*. In A172 cells, *SLC20A1* expression was significantly elevated both in low Pi loading and in the suppression of *SLC20A2*. On the other hand, *SLC20A2* expression did not change in low Pi loading-treated cells or in response to the suppression of *SLC20A1*. In SH-SY5Y cells, Pi uptake was significantly decreased by the suppression of either *SLC20A1* or *SLC20A2*. In A172 cells, Pi uptake was significantly elevated by the suppression of *SLC20A2*. In contrast, Pi uptake was significantly decreased by the suppression of *SLC20A1*. These results suggest that A172 cells have a mechanism to maintain intracellular Pi content in the physiologic range mainly through regulation by PiT-1, and that the difference in the mechanism between SH-SY5Y cells and A172 cells underlies the differential vulnerability to low Pi.

A previous study that used northern blot analysis showed a time-dependent increase in the expression of PiT-1 in response to BMP-2 in MC3T3-E1 cells, an osteogenic cell line, although the expression level of PiT-2 was not affected by BMP-2^25^. Similar findings have been demonstrated in ATDC5 chondrogenic cells, or other osteogenic cell lines, stimulated with TGF-β^26^. Another previous study showed that PiT-2 could mediate Pi uptake and calcification of cultured human vascular smooth muscle cells (VSMCs) in the absence of PiT-1, and that PiT-1 and PiT-2 could serve redundant roles in Pi-induced calcification of VSMCs^27^. In the present study, *SLC20A1* mRNA expression was significantly elevated in *SLC20A2-*suppressed A172 cells, while *SLC20A1* mRNA expression was not changed in *SLC20A2-*suppressed SH-SY5Y cells. These results indicate that Pi homeostasis may be controlled by different mechanisms with respect to the activation of PiT-1 and PiT-2, depending on the cell type, and that the degree of cellular dysfunction and/or cytotoxicity is also different even in CNS cells which express both PiT-1 and PiT-2.

Pi is one of the substrates for ATP synthesis and also plays an important role in regulating mitochondrial MP and ROS production in isolated mitochondria^28^. Therefore, the disruption of intracellular Pi homeostasis causes mitochondrial dysfunction and increased oxidative stress. Indeed, in the present study, low Pi loading lowered the mitochondrial MP and increased oxidative stress. Therefore, we investigated the effects of 5-ALA, which is thought to activate mitochondrial functioning in response to low Pi-induced cytotoxicity^29^. In the present study, 5-ALA prevented mitochondrial dysfunction and ROS production caused by low Pi loading and had no effect on mRNA expressions of *SLC20A1* and *SLC20A2*. Therefore, NaPiTs expression may not be involved in the protective effects of 5-ALA against low Pi. Our study also showed that 5-ALA suppressed cytotoxicity associated with low Pi loading *via* p38 MAPK. 5-ALA is the starting point for heme synthesis in mitochondria, and 5-ALA synthase is the rate-limiting enzyme for the production of heme. These results suggest that 5-ALA may have helped to recover mitochondrial activity in our experiment, although much research has been carried out on the detailed actions of 5-ALA in the mitochondria with respect to cellular mechanisms^16–18^.

A previous study demonstrated that 5-ALA induced expression of HO-1^16^. In mammals, HO is present in at least two isozymes, that is, an inducible type (HO-1) and a constitutive type (HO-2). HO-1 is induced by a number of stimuli such as heme, nitric oxide, heavy metals, growth factors, cytokines, modified lipids, among others^19^. HO-1 is a rate-limiting enzyme for the degradation of heme into biliverdin, carbon monoxide (CO), and free iron^30, 31^. HO-1 and its reaction products protect against various injuries^32^. HO-1 knockdown in mice results in substantially increased susceptibility to numerous toxic chemical and disease conditions associated with oxidative pathology^31^. In contrast, enhancing HO-1 activity by genetic tools or certain antioxidants has beneficial effects on cell survival. Our study demonstrated that HO-1 protein was increased by 5-ALA *via* p-38 MAPK. These results suggest that HO-1 was involved in the protective effect of 5-ALA against low Pi loading.

In IBGC, *SLC20A2* mutation is believed to reduce intracellular Pi uptake and disrupt intracellular Pi homeostasis. Low levels of intracellular Pi affect various functions such as the production of ATP, intracellular signaling and mitochondrial function. Patients with IBGC show various clinical manifestations, including psychological disorders and mental deterioration. This indicates that intracellular dysfunction such as oxidative stress occurs in the neurons vulnerable to low Pi levels in the brain. In autopsy findings, calcification is rarely seen within neurons^33^, and neuronal cell damage does not seem to be associated with calcification. From our investigation, mitochondrial dysfunction in neurons is thought to represent a crucial pathophysiological pathway in the progression of neuronal damage in patients with IBGC, although the morphological and functional abnormalities associated with this have not yet been clarified. From this viewpoint, mitochondrial dysfunction may be a therapeutic target in IBGC and 5-ALA considered a novel therapeutic candidate for neuronal protection, but not for the prevention of calcification, in IBGC.

In conclusion, our results suggest that neuronal and glial cells may exhibit different sensitivity to low Pi loading and different regulation between PiT-1 and PiT-2. These findings provide us with novel viewpoints to understand the pathophysiology in neurons and astrocytes in cases of IBGC. In addition, the activation of mitochondrial functioning may provide a new avenue with respect to clinical improvement in patients with IBGC, especially with *SLC20A2* mutation.

## Materials

### Cell culture and low Pi loading experiment

The human neuroblastoma cell line SH-SY5Y and the human glioblastoma cell line A172 were cultured in Dulbecco’s modified Eagle’s medium (DMEM) supplemented with 10% (v/v) fetal bovine serum (FBS) and kept at 37 °C in humidified 5% CO_2_/95% air. For the low Pi experiment, these cells were seeded on a 96-well plate and cultured in DMEM containing 10% FBS for 24 h. Subsequently, the medium (DMEM containing 10% FBS) was replaced with FBS-free/Pi-free DMEM (Thermo Fisher Scientific) and appropriate amounts of sodium phosphate buffer (0.1 M Na_2_HPO_4_/NaH_2_PO_4_, pH 7.4) were immediately added to produce final Pi concentrations of 0, 0.5, and 1 mM, as indicated in the figure legends. Treatment with 1 mM Pi with FBS-free DMEM was used as a control in this study.

For cytotoxicity assays, the LIVE/DEAD Cell Imaging Kit (Thermo Fisher Scientific) and Cell Count Reagent SF (Nacalai Tesque) were used, in accordance with the manufacturer’s instructions. Briefly, reagent was added to wells and the plate was incubated at 37 °C for 4 h. The optical density of formazan was detected at 450 nm by the GloMax-Multi Detection system (Promega) to calculate cell viability. A wavelength of 600 nm was used as a reference.

### siRNA transfection

For the *SLC20A1* and *SLC20A2* knockdown experiments, SH-SY5Y cells and A172 cells were transfected with a siGENOME pool targeted against human *SLC20A1* (*SLC20A1*-siRNA; L-007432-01, Dharmacon Research), *SLC20A2* (*SLC20A2*-siRNA; L-007433-02, Dharmacon Research) or siCONTROL non-targeting siRNA (NC, Dharmacon Research), using Lipofectamine RNAiMAX reagent (Life Technologies Japan), and incubated for 24 h in accordance with the manufacturer’s instructions (Thermo Fisher Scientific).

### Determination of mitochondrial membrane potential (JC-1 Staining) by fluorescence microscopy

5,5′,6,6′-tetrachloro-1,1′,3,3′-tetraethylbenzimidazolylcarbocyanine iodide (JC-1; Thermo Fisher Scientific) is a cationic dye that exhibits potential-dependent accumulation in mitochondria, indicated by a fluorescence emission shift from green (~525 nm) to red (~590 nm). Consequently, mitochondrial depolarization is indicated by an increase in the green/red fluorescence intensity ratio and can be quantified using fluorescence microscopy. Briefly, SH-SY5Y cells were incubated in a medium containing JC-1 at a final concentration of 50 µg/mL for 30 min at 37 °C, and were washed with PBS in accordance with the manufacturer’s instructions (Thermo Fisher Scientific). Samples were observed using a fluorescence microscopy system (Zeiss LSM 700, Carl Zeiss). The stock solution of JC-1 was dissolved in 100% DMSO, and the final concentration of DMSO used was <0.1%.

### ATP measurement

Intracellular ATP was measured using a luciferin–luciferase bioluminescence assay and the ‘Cell’ ATP assay reagent (Toyo Ink), in accordance with the manufacturer’s instructions. The SH-SY5Y cells were seeded on a 96-well tissue culture plate, and incubated in a CO_2_ incubator at 37 °C. The plate was incubated at room temperature for 30 min, and 100 µL ATP reagent was then added to each well. The plate was stirred for 1 min followed by incubation in a luminometer (GloMax-Multi Detection system, Promega) for 10 min at 25 °C. The relative light intensity was then measured.

### Cell culture and ROS production

To detect low Pi loading-induced intracellular ROS production, we used the redox-sensitive dyes, CellROX Green (Thermo Fisher Scientific) and MitoSOX Red (Thermo Fisher Scientific). After SH-SY5Y cells were prepared in uncoated glass-bottomed microwells, CellROX Green and MitoSOX Red were added to the cell culture to give a final concentration of 5 μM and the cells were incubated for 30 min at 37 °C, in accordance with the manufacturer’s instructions (Thermo Fisher Scientific). Samples were observed using a confocal imaging system (Zeiss LSM 700, Carl Zeiss).

### Western blotting

The treated cells were lysed by adding lysis buffer (150 mM NaCl, 10 mM Tris–HCl, 1% NP-40, 1 mM EDTA, 10 µg/mL aprotinin, 10 µg/mL leupeptin, 1% phosphatase inhibitor, 0.1 mM PMSF) and centrifuged under the conditions of 14,000 g at 4 °C for 30 min. The supernatant was collected as protein samples. The concentration of protein was determined using the Pierce BCA protein assay kit (Thermo Fisher Scientific). Samples underwent SDS-PAGE in order to separate proteins on the basis of molecular weight. SDS-PAGE was performed under constant voltage at 200 V at room temperature for 60 min. The separated proteins in polyacrylamide gel were transferred to a PVDF membrane in transfer buffer (0.3% Tris, 1.44% glycine, 20% methanol) under constant voltage at 100 V at 4 °C for 60 min. The transferred membrane was incubated in 5% skim milk (Nakarai Tesque) or 5% BSA (Wako) at room temperature for 60 min. After a blocking reaction, the membrane was incubated with primary antibodies: the mouse monoclonal antibody, β-actin (1:1000, Santa Cruz Biotechnology); rabbit polyclonal antibodies: HO-1 (1:1000, Enzo Life Sciences), p44/42 MAPK (1:1000, Cell Signaling Technology), phospho-p44/42 MAPK (1:1000, Cell Signaling Technology), p38 MAPK (1:1000, Cell Signaling Technology), and phospho-p38 MAPK (1:1000, Cell Signaling Technology), dissolved in 5% skim milk or 5% BSA at 4 °C overnight. After the primary antibody reaction, the membrane was incubated with the secondary antibody: goat anti-rabbit antibody conjugated with HRP (1:2000, Santa Cruz Biotechnology), and goat anti-mouse antibody conjugated with HRP (1:2000, Santa Cruz Biotechnology) dissolved in 3% skim milk or 3% BSA for 30 min. The membrane was incubated in ECL Prime (GE Healthcare, Buckinghamshire, UK) to generate the chemiluminescence from HRP antibodies. The chemiluminescence was detected using the LAS3000 Mini (Fujifilm). The band density was measured by ImageJ software.

### RNA preparation and qRT-PCR

Reverse transcription was performed using the SuperScript^®^ VILO cDNA Synthesis Kit (Invitrogen). The cDNA samples were used as templates for RT-PCR and qRT-PCR. The sequences of primer sets for RT-PCR are shown in Table 1. qRT-PCR was performed using the TaqMan probes (*SLC20A1*, Hs00965587; *SLC20A2*, Hs00198840; *ribosome 18 RNA*; Life Technologies) and gene-specific primers for SYBR Green on a StepOne Real-Time PCR System, in accordance with the manufacturer’s instructions (Life Technologies). The sequences of primer sets are shown in Table 2. The expression levels of mRNA were normalized to the expression levels of *ribosome 18 RNA* and *GAPDH* mRNA.

**Table 1:** Primer pairs used for the reverse transcription polymerase chain reaction (RT-PCR).

Gene	Forward	Reverse
***SLC17A1***	5′ ggctgtgccgtatgtcttct 3′	5′ cactccacccaagcaaaagc 3′
***SLC17A3***	5′ catggtcaacagcacaagcc 3′	5′ cagagaaggcagcagacaca 3′
***SLC34A1***	5′ taacgccatcctgtccaacc 3′	5′ acagagctgtcttccatggc 3′
***SLC34A2***	5′ gcatggttgactggctacct 3′	5′ tggagtttcttcggcaggac 3′
***SLC34A3***	5′ atctgctctctggacgtcct 3′	5′ agtccaactgcacgatgagg 3′
***SLC20A1***	5′ cccagacaacagatgcccat 3′	5′ aaaacgaagccacgagttgc 3′
***SLC20A2***	5′ gctccatcccacactgttca 3′	5′ actggccaatgattcccctg 3′
***18s rRNA***	5′ attcgtattgcgccgctaga 3′	5′ tcaatctcgggtggctgaac 3′

**Table 2:** Primer pairs used for the quantitative reverse transcription polymerase chain reaction (qRT-PCR).

Gene	Forward	Reverse
***HO-1***	5′ caggatttgtcagaggccctgaagg 3′	5′ tgtggtacagggaggccatcacc 3′
***NQO1***	5′ tggagtcggacctctatgcca 3′	5′ cttgtgatattccagttccccctgc 3′
***GCLC***	5′ gaagtggatgtggacaccagatg 3′	5′ ttgtagtcaggatggtttgcgataa 3′
***GCLM***	5′ ggagttcccaaatcaacccaga 3′	5′ tgcatgagatacagtgcattccaa 3′
***GAPDH***	5′ gcaccgtcaaggctgagaac 3′	5′ tggtgaagacgccagtgga 3′

### Luciferase assay

A cell suspension with Opti-MEM (Invitrogen) at the concentration of 5.0 × 10^5^ cells/mL was co-transfected with pGL4.37[luc2/ARE/Hygro] (Promega) and pAcGFP-C1 (Clontech) vectors by using the NEPA21 Super Electroporator (Nepa Gene). The transfected cells were seeded on multi-well plates of 24 wells. The medium was replaced with 1 mM Pi medium, and transfected cells were treated with 5-ALA for 30, 60, 180, and 360 min. Treated cells were lysed by adding 1× Passive Lysis Buffer (Promega) and incubated for 15 min at 4 °C. The supernatant was collected. Twenty µL of supernatant was used in the luciferase assay and 50 μL of supernatant was used to measure GFP fluorescence. For the luciferase assay, luciferin reagent (0.5 mM luciferin, 20 mM tricine, 1.1 mM MgCO_2_, 2.7 mM MgSO_4_, 33.3 mM DTT, 0.2 mg/mL coenzyme A, 0.53 mM ATP, 0.1 mM EDTA) was added to 100 μL of supernatant and samples were incubated for 10 min. The level of luminescence and GFP fluorescence was measured using the GloMax-Multi Detection system. Luciferase activity was normalized using GFP fluorescence levels.

### ^32^Pi transport assays

A Pi uptake assay was carried out on *SLC20A1*- and *SLC20A2*-suppressed cells grown to confluency in 24-well plastic plates as previously described^34^. The transport rate was expressed as nmol Pi per minute per mg protein.

### Statistical evaluation

Data are given as the mean ± standard error of the mean (SEM). The significance of differences was determined by an analysis of variance (ANOVA). Further statistical analysis for *post-hoc* comparisons was performed using the Bonferroni/Dunn test (SigmaPlot 11, Systat Software).

## Electronic supplementary material

Supplementary Data
