# Supplementary material for: Neuroprotective effect of 5-aminolevulinic acid against low inorganic phosphate in neuroblastoma SH-SY5Y cells
Source: Sci Rep. 2017 Jul 18;7:5768. doi: 10.1038/s41598-017-06406-6 (PMC5515920; doi:10.1038/s41598-017-06406-6)

(Supplementary Information)

Neuroprotective effect of 5-aminolevulinic acid against low inorganic phosphate in a neurobrastoma SH-SY5Y cells

Naoko Takase, Masatoshi Inden, Shin-ichiro Sekine, Yumi Ishii, Hiroko Yonemitsu, Wakana Iwashita, Hisaka Kurita, Yutaka Taketani & Isao Hozumi

Corresponding author: Isao Hozumi, M.D., Ph.D.

E-mail: hozumi@gifu-pu.ac.jp

Supplemental Fig S1 At 24 h after treatment of low Pi loading, mRNA expressions of *SLC20A1* and *SLC20A2* were analyzed by the TaqMan-based qPCR assay. Data are normalized to the amount of *18s rRNA*, and results are expressed as the fold increase compared with that at control (mean ± SEM; *n* = 3).

Supplemenrtal Fig S1

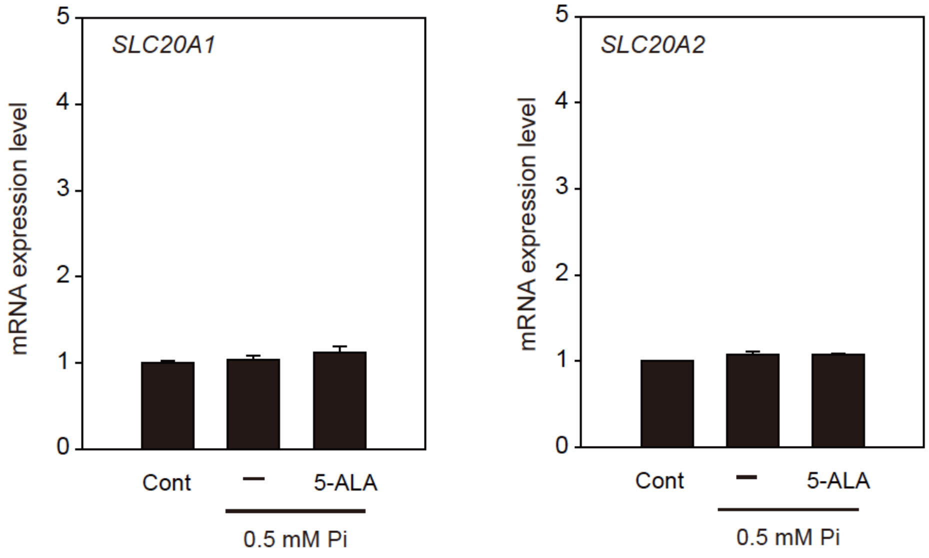

Supplement: Supplementary file 1 — Supplementary Data [file 41598_2017_6406_MOESM1_ESM.pdf]
